# *hsa_circ_0000231* Promotes colorectal cancer cell growth through upregulation of *CCND2* by *IGF2BP3/miR-375* dual pathway

**DOI:** 10.1186/s12935-022-02455-8

**Published:** 2022-01-15

**Authors:** Wei Zhang, Bo Wang, Yilin Lin, Yang Yang, Zhen Zhang, Quan Wang, Haoran Zhang, Kewei Jiang, Yingjiang Ye, Shan Wang, Zhanlong Shen

**Affiliations:** 1 Department of Gastroenterological Surgery, Peking University People’s Hospital, Beijing, 100044 People’s Republic of China; 2 Laboratory of Surgical Oncology, Peking University People’s Hospital, Beijing, 100044 People’s Republic of China; 3 Beijing Key Laboratory of Colorectal Cancer Diagnosis and Treatment Research, Peking University People’s Hospital, Xizhimen South Street, Xicheng, Beijing, 100044 People’s Republic of China

**Keywords:** Colorectal cancer, CircRNA, *IGF2BP3*, *miR-375*, *CCND2*

## Abstract

**Background:**

Circular RNAs (circRNAs) have emerged as vital regulators of the initiation and progression of diverse kinds of human cancers. In this study, we explored the role of *hsa_circ_0000231* and its downstream pathway in CRC.

**Methods:**

The expression profile of circRNAs in 5 pairs of CRC tissues and adjacent normal tissues were analyzed by Microarray. Quantitative real-time PCR and in situ hybridization and Base Scope Assay were used to determine the level and prognostic values of *hsa_circ_0000231*. Then, functional experiments in vitro and in vivo were performed to investigate the effects of *hsa_circ_0000231* on cell proliferation. Mechanistically, fluorescent in situ hybridization, dual luciferase reporter assay, RNA pull-down and RNA immunoprecipitation experiments were performed to confirm the interaction between *hsa_circ_0000231* and *IGF2BP3* or has_*miR-375*.

**Results:**

We acquired data through circRNA microarray profiles, showing that the expression of *hsa_circ_0000231* was upregulated in CRC primary tissues compared to adjacent normal tissues, which was indicated poor prognosis of patients with CRC. Functional analysis indicated that inhibition of *hsa_circ_0000231* in CRC cell lines could suppress CRC cell proliferation as well as tumorigenesis in vitro and in vivo. The mechanistic analysis showed that *hsa_circ_0000231* might, on the one hand, act as a competing endogenous RNA of *miR-375* to promote cyclin D2 (*CCND2*) and, on the other hand, bind to the *IGF2BP3* protein to prevent *CCND2* degradation.

**Conclusions:**

The findings suggested that *hsa_circ_0000231* facilitated CRC progression by sponging *miR-375* or binding to *IGF2BP3* to modulate *CCND2*, implying that *hsa_circ_0000231* might be a potential new diagnostic and therapeutic biomarker of CRC.

**Supplementary Information:**

The online version contains supplementary material available at 10.1186/s12935-022-02455-8.

## Background

Colorectal cancer (CRC) is the third most commonly diagnosed cancer in men and the fourth most commonly diagnosed cancer in women worldwide [1]. The annual new cases of CRC account for its third place among malignant tumors, and the related deaths account for the fourth place [2]. CRC was the fifth most commonly diagnosed cancer and the fifth most common cause of death by cancer in China in 2014, with an age-standardized incidence rate of 17.52 per 100,000 and age-standardized mortality rate of 7.91 per 100,000 [3].

CRC has become a major disease seriously threatening human health and posing a huge social and economic burden [4]. The treatment of CRC mainly adopts a comprehensive treatment mode centered on surgery, but the curative effect is still unsatisfactory. The molecular mechanism underlying the development of CRC is not completely clear, which is one of the important reasons for its high incidence and poor prognosis [5]. Recent studies have shown the importance of a new family of noncoding RNAs, circular RNAs (circRNAs), in the molecular regulation of tumorigenesis and progression. They have a covalently closed circular structure at the 3′-untranslated region (UTR) and 5′-UTR, are widely and diversely present in eukaryotic cells, and have an endogenous RNA molecule that regulates gene expression [6–8]. The circRNA functions mainly via the endogenous RNA (ceRNA) mechanism, that is, circRNA acts as a "molecular sponge" and competitively binds to microRNA (miRNA) through a miRNA response element (MRE) to inhibit miRNA function. MiRNAs degrade or inhibit the expression of target genes through the RNA-induced silencing complex (RISC), which relies on the Argonaute2 (*AGO2*) protein [9].

MicroRNAs (miRNAs) are small noncoding RNAs with a size of 18–25 nucleotides, which function as post-transcriptional regulators of target mRNAs [10]. The increase in the expression of oncogenic miRNAs in cancer leads to the downregulation of tumor-suppressive genes. In contrast, the decrease in the expression of tumor-suppressive miRNAs enhances the expression of oncogenes. The findings indicated that miRNAs participated in the tumorigenesis and progression of various cancers, including colorectal cancer [11–13]. Also, circRNAs could bind to RNA-protective proteins [14]. However, the biological functions of most circRNAs in the pathogenesis and progression of CRC and the underlying mechanisms remain largely unclear.

The present study found 425 significantly differentially expressed circRNAs in CRC tissues. The *hsa_circ_0000231* upregulation was further discovered to sponge *miR-375* and *IGF2BP3* and hence regulate CRC progression. The *hsa_circ_0000231* is vital in the development of CRC and participates in the molecular regulation of CRC by regulating the cell cycle.

## Methods

### CircRNA microarray

Five pairs of CRC tumor tissues and corresponding adjacent noncancerous tissues were used for circRNA microarrays. The specimens were obtained from patients undergoing surgery in the Peking University People’s Hospital in 2014; detailed information is shown in Table 1.

**Table 1:** Clinicopathological features for circRNA microarray

	Numbers	Percent (%)
Gender		
Male	4	80
Female	1	20
Age at diagnosis		
≤ 60	2	40
> 60	3	60
Tumor size (cm)		
≤ 5	5	100
> 5	0	0
Differentiation		
Well-moderate	0	0
Poor	0	0
Depth of invasion		
T1–T2	0	0
T3–T4	0	0
Lymph node metastasis		
No	2	40
Yes	3	60
TNM stage		
I–II	2	40
III–IV	3	60

### Patients and samples

160 CRC patients who were diagnosed and underwent surgery in Peking University People’s Hospital between 2014 and 2017 were included in this study. Fresh colorectal tumor tissues and matched normal colorectal mucosa tissues were obtained from all the 160 patients. The specimens were obtained and immediately frozen in liquid nitrogen and stored at − 80 °C until RNA or protein extraction. This study was performed according to the recommendations in the Guide for the Chinese Ethics Review Committees. The protocol was approved by the Ethics Committee of Peking University People’s Hospital. Written informed consent was obtained from each subject. The animal experiment was carried out under ethics approval of Peking University People’s Hospital.

### Cell culture

Human colorectal cancer SW480, SW620, HCT116, HT29, RKO, LS174T, COLO-215, HCT8 and LoVo cell lines were purchased from American Type Culture Collection (Manassas, USA). Human normal intestinal epithelial cells NCM460 were purchased from INCELL Corporation LLC (San Antonio, USA). HCT116, HT29, RKO, LoVo, COLO-215, HCT8 and NCM460 cells are cultured in RPMI-1640. LS174T cells were cultured in DMEM. SW480 and SW620 were cultured in Leibovitz's L-15 medium. All cells were cultured at 37˚C containing 5% CO_2_. All medium were supplemented with 10% fetal bovine serum (FBS, Gibco, USA).

### Cellular fluorescence in situ hybridization

The cells were fixed with 4% paraformaldehyde for 15 min and washed with phosphate-buffered saline (PBS) three times for 5 min each time. Further, they were permeabilized with 0.5% Triton X-100 at room temperature for 20 min and washed with PBS three times for 3 min each time. Then, pepsin freshly diluted with 3% citric acid was added and digested at room temperature for 15 min. Subsequently, the nucleic acid fragment was exposed, rinsed with PBS, mixed with 20 μL of a pre-hybrid solution, and pre-hybridized at 50 °C for 2–4 h. The *hsa_circ_0000231* or miR375-specific probe hybridized at a constant temperature of 50 °C. Hybridization was carried out using SSC at 37 °C, biotinylated mouse anti-digoxigenin was added dropwise, the fragment was washed with PBS three times for 3 min each time, and the excess solution was absorbed with the absorbent paper. After adding DAPI stain for 10 min, the specimen was subjected to nuclear staining and washed three times with PBS for 3 min each time; the excess solution was absorbed by the absorbent paper. The specimen was sealed with a liquid containing a fluorescent quencher, and the image was observed under a fluorescence microscope.

### BaseScope assay

BaseScope assay was performed following the manufacturer's protocols (Advanced Cell Diagnostics, CA, USA). The tissues were sectioned at 5-μm thickness, placed onto Superfrost Plus slides (Fisher Scientific, Loughborough, UK), and allowed to dry overnight at 25 °C. The sections were then baked at 60 °C for 1 h before deparaffinized in xylene (twice for 5 min) and ethanol (twice for 2 min), and then dried by baking at 60 °C for 2 min. Subsequently, hydrogen peroxide was applied for 10 min at 25 °C, target retrieval was performed for 15 min at 100 °C, and RNAscope Protease III was applied at 40 °C for 30 min. The samples were rinsed twice in distilled water between treatments. BaseScope probes (Mm-1700024F13Rik, cat#709881) with positive controls (Hs-PPIB, cat # 701031; DapB, cat # 701011) were then applied, and the samples were incubated for 2 h at 40 °C in a HybEZ oven and then with reagents AMP0 (30 min at 40 °C), AMP1 (15 min at 40 °C), AMP2 (30 min at 40 °C), AMP3 (30 min at 40 °C), AMP4 (15 min at 40 °C), AMP5 (30 min at 25 °C), and AMP6 (15 min at 25 °C). The slides were rinsed with wash buffer (twice for 2 min) between AMP incubation steps. Finally, they were treated with Fast Red for 10 min at 25 °C in the dark, counterstained with Gill's hematoxylin, dried for 15 min at 60 °C, and mounted in Catamount permanent mounting medium (Vector Labs, CA, USA).

### RNA extraction and quantitative real-time polymerase chain reaction

Total RNA from cell lines and tissue samples was extracted using TRIzol (Invitrogen, USA) following the manufacturer’s instructions. For the plasma, the total RNAs were extracted using an mirVana PARISTM microRNA extraction kit (ABI, USA) following the manufacturer’s protocols. For lncRNA quantification, GAPDH was used as internal control, and PrimeScript RT Master Mix (QIAGEN, Germany) was used for reverse transcription and real-time polymerase chain reaction (PCR). For circRNA expression detection, RNase R was added to remove linear RNA after the sample RNA was extracted, and then random primers were reverse transcribed into cDNA for further amplification. For the design of circRNA amplification primers, this study adopts the design method of trans circularization site primers. The primer sequences of all genes are shown in Additional file 1: Table S1. All reactions were performed in triplicate. The fold change for each gene relative to the control group was calculated using the 2^−ΔΔCt^ method.

### Lentiviral short hairpin RNA particles

Recombinant lentiviral particles expressing *hsa_circ_0000231* and normal control (NC) were obtained from GenePharm Co., Ltd. (Shanghai, China). SW480 cells were grown to approximately 40% confluence and infected with lentiviral particles in complete medium for 48 h. They were co-treated with the cationic polymer polybrene (8 μg/ml in water) to increase the infection efficiency. Neither shRNA nor polybrene affected cell viability. Further, shRNA had no off-target effects, and did not affect cell adherence, shape, or viability at the indicated multiplicity of infection.

### Cell transfection

For in vitro studies, siRNA interference sequences targeting *hsa_circ_0000231* and the best transcript of *ARHGAP12* which *hsa_circ_0000231* located at—mRNA (NM_018287) normal control (si-NC) were designed and synthesized (Ribobio, Guangzhou, China) to interfere with the expression of *hsa_circ_0000231* or mRNA, which named si-circ_0000231 or si-mRNA, and a final concentration of 50 nM was used for transient transfection. Similarly, mimics-NC and *miR-375* mimics were designed and synthesized (Ribobio, Guangzhou, China) to overexpress *miR-375*. Lipofectamine 3000 (Invitrogen, CA, USA) was used for transfection following the manufacturer’s protocols.

For in vivo assays, the *hsa_circ_0000231* overexpression cell line was used. The *hsa_circ_0000231* gene was cloned into a lentivirus vector LV-GFP-Puro, and SW480 cells were used for infection. Stable transfection cells were established by puromycin antibiotic selection for 7 days, with a concentration of 2.5 µg/ml. The *hsa_circ_0000231*-overexpressing cells and control cells were named LV-*hsa_circ_0000231*(LV-circ) and LV-NC, respectively.

Transfection and grouping of cells: Cell transfection were performed in strict accordance with the instructions of Invitrogen's Lipofectamine 3000 Transfection Reagent when the cytoplasm was inoculated with a confluence of about 80%. The Lipofectamine 3000 reagent was diluted with OPTI-MEM culture medium and mixed. The DNA expression plasmid to be transfected was diluted with OPTI-MEM culture medium and mixed with P3000 reagent. The diluted DNA expression plasmid was added in equal volume to each dilution of Lipofectamine 3000 reagent and incubated at room temperature for 5 min. The DNA–liposome mixture was added to the cell suspension and carefully mixed. The culture was continued in a 5% CO_2_ incubator at 37 °C.

### Cell proliferation assay

SW480 and SW620 cells (3 × 10^3^ cells) were seeded in complete medium in 96-well plates and infected with *hsa_circ_0000231* siRNA. The cell proliferation assay was performed with a Cell Counting Kit 8 (CCK8) following the manufacturer’s protocol, and cell proliferation was detected after 0, 24, 48, 72, and 96 h. The cells in each group were tested for five replicates. The cell proliferation was evaluated by the CCK-8 method using a microplate reader (Molecular Devices, CA, USA) following the manufacturer’s protocols to measure the absorbance.

For the colony formation assay, the transfected cells were seeded into each well of a 6-well plate on day 0 and then incubated for another 14 days. Then, the wells were fixed with 4% paraformaldehyde and stained with 0.1% crystal violet. The colonies so formed were counted and analyzed using Image J software.

### Western blot analysis

RIPA lysis buffer (Solarbio, Beijing, China) to extract total protein. The proteins were quantified by the bicinchoninic acid (BCA) assay kit (Solarbio, Beijing, China). Add SDS loading buffer to the extracted total protein, and then boil it in 100° water for five minutes for subsequent experiments. The obtained protein was electrophoresed in 12% sodium dodecyl sulfate–polyacrylamide gel electrophoresis (SDS-PAGE). PVDF membrane was used for electroporation. The protein electrophoresis was performed at a stable voltage of 100 V, and the electroporation was performed at a stable current of 300 mA for 90 min. After electroporation, soak the PVDF membrane in 5% skimmed milk and place it on a shaker for half an hour. Then add 5 ml of CCND2 (1:1000), IGF2BP3 (1:1000) (Abcam, CA, USA), RB (1:500), and GAPDH (1:5000) (Cell Signaling Technology, MA, USA) rabbit-derived primary antibody to the PVDF membrane and incubate overnight at 4°. After incubating overnight, collect the primary antibody, add Tris-Buffered Sal ine Tween 20 (TBST) and wash three times for 15 min each time. Add goat anti-rabbit (Solarbio, Beijing, China, 1:5000) and incubate for 1 h, then continue to wash with TBST three times. Finally, add electrochemiluminescence (Solarbio, Beijing, China) liquid to expose in the exposure instrument. The antibodies used in the experiments are shown in Additional file 2: Table S2.

### Luciferase reporter assay

The *hsa_circ_0000231* and *CCND2* fragments containing two putative wild-type or mutated *miR-375*-binding sites were amplified by PCR and cloned downstream of the luciferase gene in the pGL3 vector (Promega, WI, USA). The constructed reporter vectors were verified by sequencing. Luciferase reporter assays were performed by transiently co-transfecting SW480 cells in 24-well plates with the reporter vectors, *miR-375*, and the Renilla luciferase construct using Lipofectamine 2000 (Invitrogen, MA, USA). After 48-h transfection, the cells were harvested, and luciferase activity was measured using a dual-luciferase reporter assay system (Promega) and normalized to that of Renilla luciferase.

### Biotin-labeled RNA pull-down and mass spectrometry analysis

Biotin-labeled RNA for the linear sequence of *hsa_circ_0000231* and *CCND2* was generated by an in vitro transcription reaction with the Biotin RNA Labeling Mix (Roche, Mannheim, Germany) and T7 RNA polymerase (Roche), and then treated with RNase-free DNase I (TaKaRa, Japan). After incubation with the oligonucleotide targeting circular junction, the liner probe was circularized using T4 RNA ligase I and treated with RNase R. After purification with a RNeasy Mini Kit (Qiagen, Inc., CA, USA), the biotin-labeled RNA probe (3 μg) was incubated with cell extracts from CRC cells at room temperature for 2 h and treated with 35 μL of Streptavidin C1 magnetic beads (Invitrogen) for 1 h. After washing, the retrieved protein was detected by Western blot or mass spectrometry analysis (CapitalBio Technology, Beijing, China).

### RNA immunoprecipitation

RNA immunoprecipitation (RIP) was conducted with a Magna RIP kit (Millipore, MA, USA) following the manufacturer’s instructions. SW480 cells were harvested 48 h after the transfection of *miR-375* mimics or miR-NC and lysed in complete RNA lysis buffer. The cell lysates were incubated with magnetic beads conjugated with anti-AGO2 (Millipore) or negative control immunoglobulin antibody (Millipore) at 4 °C for 4 h. The beads were washed with wash buffer. Then, immunoprecipitated RNA and protein were purified and enriched to detect the target RNAs and AGO2 using qRT-PCR and Western blot analysis.

### Nude mouse model of ectopic tumors

This study was conducted in accordance with the ethical standards, the Declaration of Helsinki, and national and international guidelines, and was approved by the authors’ institutional review board, which adheres to generally accepted international guidelines for animal experimentation.

BALB/c nude (nu/nu) mice, aged 6 weeks old were purchased from Beijing Weitong Lihua Experimental Animal Technology Co., Ltd. Tumors were generated by the subcutaneous injection of 2 × 10^6^ SW480 cells infected with *hsa_circ_0000231*-overexpressing cells or control lentivirus particles and suspended in 50 L of PBS into the dorsal region near the thigh. Five mice were included in each group. The mice were then weighed and assessed for tumor size every 7 weeks by measuring the tumor length and width. The mice were sacrificed after cervical dislocation, the skin was wiped with 75% ethanol, the abdominal cavity was cut open, and the liver tissues were cut. PMSF (Sigma, MA, USA) (20ug/2 ml) were added into the precooled cracking fluid. Take 2 ml pre-cooled cracking buffer, quickly add it to homogenizer, and fully grind it under ice bath condition. The samples were centrifuged at 4 ℃ for 15000 rpm for 10 min. Take the crude extract of supernatant tissue protein to a new 1.5 mL centrifuge tube. The protein was quantified and stored at − 80 ℃.

### Statistical analysis

The data generated in this study were all analyzed using SPSS22.0 statistical software. The measurement data were expressed as mean ± standard deviation (x ± s). The two groups were compared using the *t* test for statistical analysis. The count data were analyzed using the *χ*^2^ test. The survival rates were evaluated using the Kaplan–Meier method and tested using the log-rank test. The effects of clinical variables on the overall survival of patients with CRC were determined by univariate and multivariate Cox proportional hazards regression models. Age, T stage, N stage, clinical stage of distant metastasis, and expression of *hsa_circ_0000231* were adjusted for variable analysis in the multivariate Cox proportional hazards regression model. The correlation between groups was analyzed by Pearson correlation, and a *p* value < 0.05 was used as a criterion for statistically significant differences. (Additional file 3: Tables S3, Additional file 4: S4).

## Results

### Expression of *hsa_circ_0000231* was upregulated in CRC specimens and associated with the progression and poor prognosis of patients with CRC

**Fig. 1:**
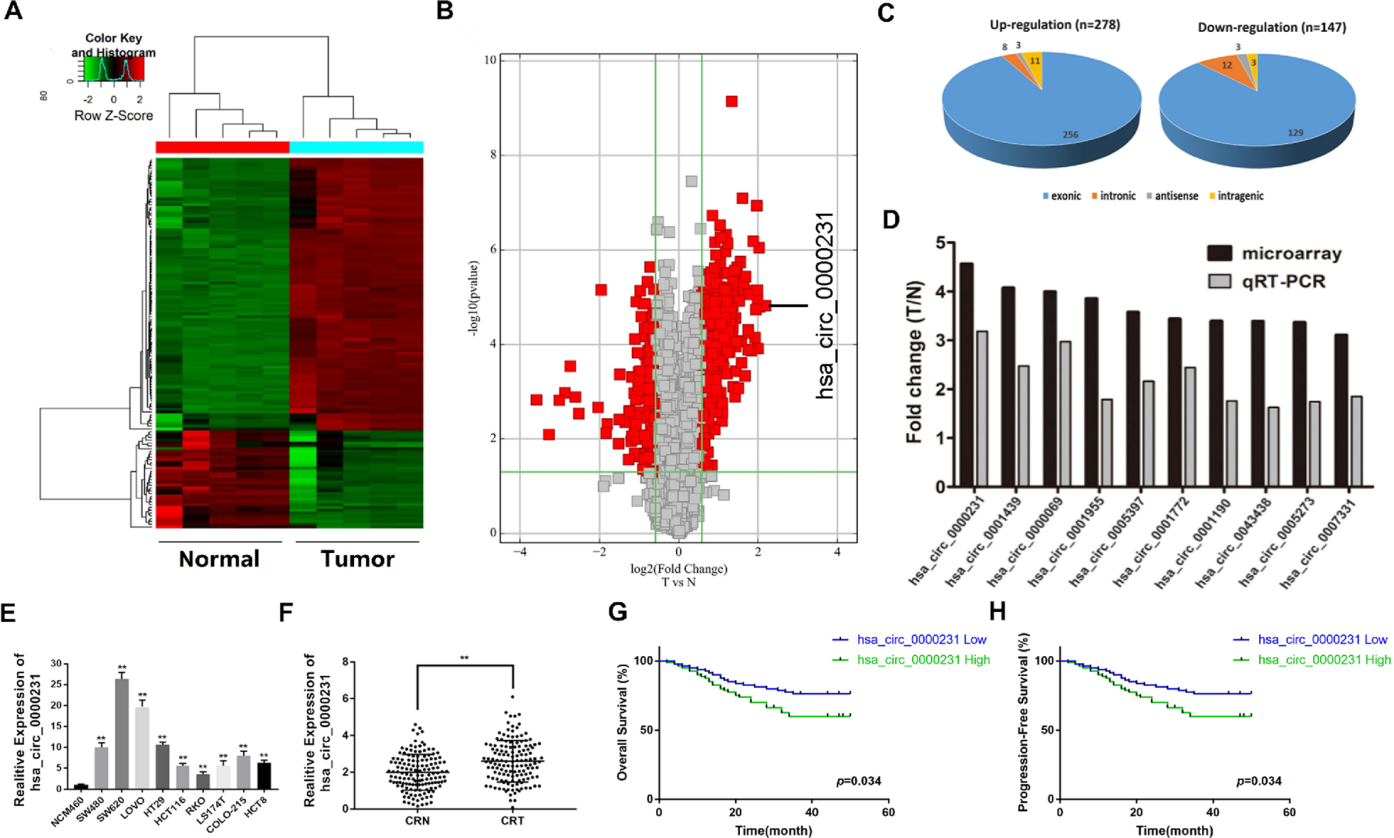
Expression profiles of circRNA and the correlation between *hsa_circ_0000231* and poor prognosis of CRC. **A** The cluster heat maps displayed the increased and decreased circRNAs. Each column indicates a sample while each row indicates an individual circRNA. The red and green strips represent high and low expression, respectively. **B** The volcano plot visualizes the expression of circRNA between CRC tissues and adjacent. The red dot on the left represents the significantly high expression of circRNAs in adjacent normal tissue. The red dot on the right represents the significantly high expression of circRNAs in CRC. **C** Classification of differentially expressed circRNA between CRC tissues and adjacent. **D** qRT-PCR assay was used to verify the results of circRNA microarray assay. The result shows that *hsa_circ_0000231* has the largest fold change. **E** Relative expression of hsa_circ_0000231 in CRC cell lines was determined by qRT-PCR. The results showed that the expression of *hsa_circ_0000231* in CRC cell lines were significantly higher than that in NCM460 cells. **F** Relative expression of *hsa_circ_0000231* in CRC tissues (CRT) and adjacent normal tissues (CRN) was detected by qRT-PCR (n = 160). The result showed that the expression of *hsa_circ_0000231* was higher in tumor tissues than in adjacent normal tissues. **p < 0.001. **G** Kaplan–Meier survival curve of overall survival in 160 patients with CRC according to the *hsa_circ_0000231* expression. Patients were divided into high expression and low expression group by median expression. Patients with high expression of *hsa_circ_0000231* in CRC had significantly shorter OS, with statistically significant differences. **H** Kaplan–Meier survival curve of progression-free survival in 160 patients with CRC according to the *hsa_circ_0000231* expression. Patients were divided into high expression and low expression group by median expression. Patients with high expression of *hsa_circ_0000231* in CRC had significantly lower DFS, with statistically significant differences

Arraystar circular RNA chip experiments were conducted in paired CRC tissues and para-cancerous tissues from five patients with CRC to understand the expression profiles of circRNA in CRC. With a cutoff criteria of fold change > 1.5 and *P* < 0.05, 425 circRNAs were found to be differentially expressed, of which 278 were upregulated and 147 were downregulated (Fig. 1A, B). Besides, the exon-related circRNAs were the maximum according to the classification analysis of circRNAs (Fig. 1C, Additional file 5: Table S5). Further, *hsa_circ_0000231* was the most upregulated (4.557-folds) circRNA, which was spliced from *ARHGAP12* located at chr10:32197099–32199491 and finally formed a circular transcript of 794 nucleotides according to the annotation of circBase (http://www.circbase.org/).

The 10 cirRNAs with the most up-regulated expression in colorectal cancer were selected, and quantitative PCR detection was performed in 10 randomly selected CRC tissues. These results were consistent with the chip results, indicating that the chip analysis results were credible (Fig. 1D). Further quantitative PCR was performed to detect the expression level of hsa_circ_0000231 in 9 CRC cell lines (SW480, SW620, LoVo, HT29, HCT116, RKO, LS174T, COLO-215 and HCT8) and NCM460 cell derived from normal human colorectal epithelial cells. The results showed that the expression of *hsa_circ_0000231* in CRC cell lines were significantly higher than that in NCM460 cells. (Fig. 1E). Morever, the expression of *hsa_circ_0000231* in colon cancer tissues and adjacent normal tissues in 160 patients was detected by quantitative PCR. The result showed that the expression of *hsa_circ_0000231* was higher in tumor tissues than in adjacent normal tissues by 1.39 times (Fig. 1F).

In order to further explore the relationship between *hsa_circ_0000231* and the prognosis of CRC, the Kaplan–Meier curve was used to analyze the relationship of *hsa_circ_0000231* with overall survival (OS) and disease-free survival (DFS) in patients with CRC. Patients with high expression of *hsa_circ_0000231* in CRC had significantly shorter OS and lower DFS, with statistically significant differences (Fig. 1G, H).

### *Hsa_circ_0000231* promoted CRC cell proliferation and regulated cell cycle and

**Fig. 2:**
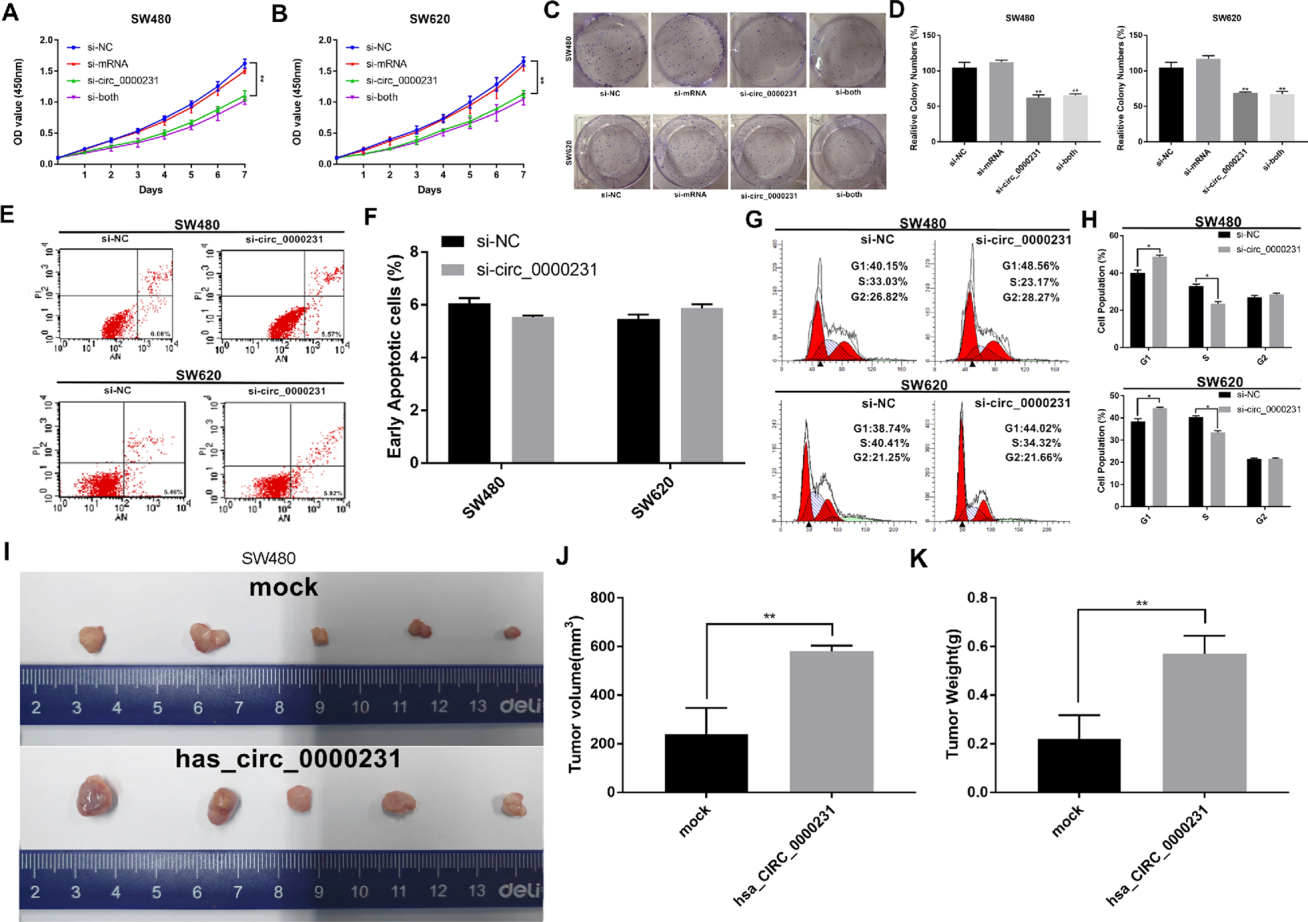
*hsa_circ_0000231* promotes cell proliferation and tumor growth in vitro and in vivo in CRC. **A** and **B** CCK8 assay was performed to detect the effect of *hsa_circ_0000231* on cell proliferation. The number of cell proliferation decreased significantly in SW480 cells or SW620 cells in the *si_circ_0000231* and *si_circ_0000231* combined with si-mRNA (NM_018287) groups (si-both) compared with the control group. **C** and **D** Colony formation assays were executed to detect the proliferation of cells transfected with indicated vectors. The result showed that the downregulation of *hsa_circ_0000231* could markedly reduce the cell cloning capabilities of SW620 and SW480 compared with the negative control group (si-NC). **E** and **F** Apoptosis rate was analyzed by flow cytometry after downregulation of *hsa_circ_0000231*. The results showed that knocking down *hsa_circ_0000231*, there was no significant difference in apoptosis of the two groups of cells. **G** and **H** The cell cycle progression was analyzed by flow cytometry after downregulation of *hsa_circ_0000231*. The cell cycle detection by flow cytometry revealed that the percentage of G1 cells obviously increased after inhibiting the expression of *hsa_circ_0000231* in SW480 and SW620 cells. **I** Images of xenograft tumors of each group (n = 5). **J** The difference in tumor volume in different intervention groups. The result showed that the tumor volume in the *hsa_circ_0000231* group was significantly higher than that in the mock group. **K** The difference in tumor weight in different intervention groups. The result showed that the tumor weight in the *hsa_circ_0000231* group was significantly higher than that in the mock group. Data were showed as mean ± SD, *p < 0.05, **p < 0.001

The effect of *hsa_circ_0000231* on cell proliferation was detected using the CCK-8 assay. The number of cell proliferation decreased significantly in SW480 cells or SW620 cells in the *si_circ_0000231* and *si_circ_0000231* combined with si-mRNA (NM_018287) groups (si-both) compared with the control group (p < 0.05) (Additional file 6: Fig. S1, Fig. 2A, B). Then colony formation assays further displayed that the downregulation of *hsa_circ_0000231* could markedly reduce the cell cloning capabilities of SW620 and SW480 compared with the negative control group (si-NC) (p < 0.001) (Fig. 2C, D).

In order to further verify whether the decrease in cell number is caused by decreased proliferation or increased apoptosis, we conducted a cell apoptosis test. The flow cytometry analysis with Annexin V/PI double staining showed no significant difference in the apoptotic rate of SW480 and SW620 cells transfected with each group of siRNAs in the *si-circ_0000231* group compared with the negative control (si-NC) group (Fig. 2E, F).

The above results suggest that down-regulating the expression of *circ_0000231* may inhibit cell proliferation. For this reason, we conducted a cell proliferation test by flow cytometry. The cell cycle detection by flow cytometry revealed that the percentage of G1 cells obviously increased after inhibiting the expression of *hsa_circ_0000231* in SW480 and SW620 cells. These results indicated that the knockdown of *hsa_circ_0000231* induced G0/G1 phase arrest in CRC cell lines (*p* < 0.05) (Fig. 2G, H).

### *Hsa_circ_0000231* facilitated the tumorigenesis and proliferation of CRC cells in vivo

SW480 cells were stably transfected with the overexpression or mock vector. The *hsa_circ_0000231* overexpression lentivirus and negative control were obtained from Gene Pharm Co., Ltd. (Shanghai, China). Then cells were injected subcutaneously into female nude mice to determine the effects of *hsa_circ_0000231* on tumor growth in vivo. The results showed that in the overexpression *hsa_circ_0000231* group, the tumor volume and tumor weight were significantly higher than those in the control group. (Fig. 2I–K). These results confirmed the oncogenic role of *hsa_circ_0000231* in the development of CRC, suggesting the involvement of *hsa_circ_0000231* in the progression of CRC.

### *CCND2* might be a downstream target of *hsa_circ_0000231* in CRC

In order to further explore the downstream regulatory mechanism of *hsa_circ_0000231*, we conducted further analysis on SW480 cells transfected with *si_circ_0000231*. The PCR chip (Cell Cycle PCR array, Qiagen) testing of cell cycle–related genes found that the expression of 18 genes of the 84-cell cycle–related genes more than doubled compared with the control group. The 10 genes with the most decreased expression were depicted in Additional file 7: Fig. S2A. The downregulation of *CCND2*, *CCND1*, *CDK6*, and *CDKN3* was the most obvious (the downregulation factor was more than three times). The qPCR experiment further verified that the mRNA expression trends of *CCND2*, *CCND1*, *CDK6* and *CDKN3* were consistent with the cell cycle chip results (Additional file 7: Fig. S2B). The above studies have found that inhibiting the expression of *hsa_circ_0000231* can significantly inhibit the expression of cell cycle-related gene mRNA. In addition, through further analysis, it was found that *CCND2* mRNA had the largest fold change. Hence, the follow-up of this research focuses on the exploration of the mechanism between *hsa_circ_0000231* and *CCND2* in the occurrence and progression of CRC.

The expression of *hsa_circ_0000231* was interfered by transfection of siRNA, and the expression of *CCND2* was significantly reduced by qRT-PCR (Additional file 7: Fig. S2C). Then, the expression of *CCND2* in 160 CRC cohorts was detected by qPCR. The results showed that the expression of *CCND2* was notably higher in tumor tissues than in adjacent normal tissues (Additional file 7: Fig. S2D). The survival analysis was performed to reveal that patients with high expression of *CCND2* had significantly shorter OS (Additional file 7: Fig. S2E). Besides, the expression of *CCND2* positively correlated with *hsa_circ_0000231* (Additional file 7: Fig. S2F). In a study on nude mice, the Western blot analysis revealed that the expression of *CCND2* in *hsa_circ_0000231*-overexpressing tumor tissues was much higher than that in the mock group (Additional file 7: Fig. S2G, H).

### *Hsa_circ_0000231* functioned as a sponge for *miR-375*

**Fig. 3:**
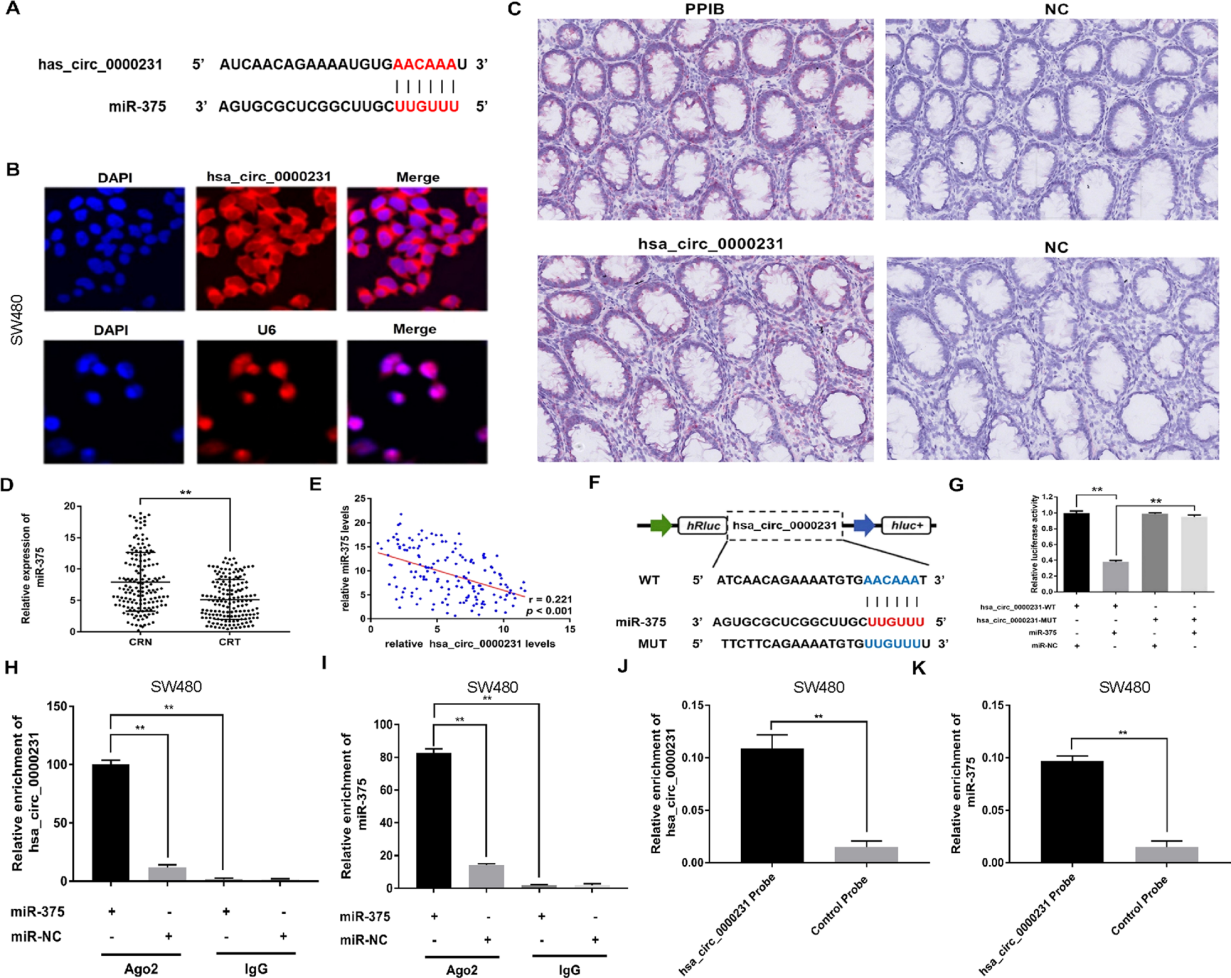
*hsa_circ_0000231* functions as a sponge for *miR-375* in CRC. **A** The *miR-375* binding site on *hsa_circ_0000231* predicted by targetScan and miRanda. **B** FISH assay was performed to observe the cellular location of *hsa_circ_0000231* (red) in cells (magnification, × 200, scale bar, 50 μm). **C** BaseScope assay was performed to observe the cellular location of *hsa_circ_0000231* (red) in CRC tissues (magnification, × 100, scale bar, 100 μm). It was found that most of the expression of hsa_circ_0000231 was located in the cytoplasm. **D** Relative expression of *miR-375* in CRC tissues (Tumor) and adjacent non-tumor tissues (Normal) was tested by qRT-PCR (n = 160). The result indicated that the expression of *miR-375* was markedly downregulated in CRC tissues compared with adjacent nontumor tissues. **E** Pearson correlation analysis of *hsa_circ_0000231* and *miR-375* expression in 160 CRC tissues. The result indicated that the expression of *miR-375* negatively correlated with *hsa_circ_0000231. F* Schematic illustration of *hsa_circ_0000231*-WT and *hsa_circ_0000231*-Mut luciferase reporter vectors. **G** The relative luciferase activities were detected in SW480 cells after transfection with *hsa_circ_0000231*-WT or *hsa_circ_0000231*-Mut and *miR-375* mimics or miR-NC, respectively. **H** and **I** Anti-AGO2 RIP was executed in SW480 cells after transfection with miR-375 mimic or miR-NC, followed by western blot and qRT-PCR to detect AGO2 protein, *hsa_circ_0000231* and *miR-375*, respectively. **J** and **K** RNA pull-down was executed in SW480 cells, followed by qRT-PCR to detect the enrichment of *hsa_circ_0000231* and *miR-375*. Data were showed as mean ± SD, *p < 0.05, **p < 0.001

The potential targets of *hsa_circ_0000231* were predicted using miRNA target prediction software made by Arraystar according to the TargetScan and miRanda database to elucidate the molecular mechanism underlying *hsa_circ_0000231* regulating *CCND2*, given that circRNAs might act as a sponge for microRNAs further modulating downstream targets. The results showed that *hsa_circ_0000231* possessed a conserved target site of *miR-375* with a high score (Fig. 3A). Considering that circRNAs could serve as miRNA sponges in the cytoplasm, fluorescence in situ hybridization and BaseScope Assay were performed in CRC cells and tissues to observe the subcellular localization of *hsa_circ_0000231*. Most of *hsa_circ_0000231* located in the cytoplasm (Fig. 3B, C). Then, the levels of *miR-375* were examined in 160 pairs of CRC tissues and adjacent noncancerous tissues. The results indicated that the expression of *miR-375* was markedly downregulated in CRC tissues compared with adjacent nontumor tissues (Fig. 3D), and the expression of *miR-375* negatively correlated with *hsa_circ_0000231* (Fig. 3E). Therefore, it was supposed that *hsa_circ_0000231* might serve as a competing endogenous RNA (ceRNA) for *miR-375*.

The dual-luciferase reporter assay was applied in SW480 cells to confirm the bioinformatics prediction analysis. The full-length *hsa_circ_0000231*-WT and mutant version without *miR-375*-binding sites were subcloned into luciferase reporter vector psiCHECK2 (Fig. 3F). The results indicated that *miR-375* mimics could significantly decrease the luciferase activity in the WT group but in the not mutant one (Fig. 3G), suggesting a direct interaction between *hsa_circ_0000231* and *miR-375*.

It is known that miRNAs regulate target gene expression by binding to AGO2, the key component of RISC. Therefore, RIP assay was performed in SW480 cells to detect RNA bound to AGO2 protein. Indeed, AGO2, *hsa_circ_0000231* and *miR-375* were all efficiently pulled down by anti-AGO2 antibodies compared with IgG. Moreover, both *hsa_circ_0000231* and *miR-375* were significantly enriched in cells transfected with *miR-375* mimics compared with the miR-NC group (Fig. 3H, I).

A circRNA pull-down assay with specific biotin-labeled *hsa_circ_0000231* probes were performed to further verify the binding effect of *hsa_circ_0000231* and *miR-375*. A specific enrichment of *hsa_circ_0000231* and *miR-375* was detected by qRT-PCR in the *hsa_circ_0000231* probe group compared with the control probe group (Fig. 3J, K).

### *MiR-375* suppressed the growth of CRC cells in vitro and in vivo

The above results indicate that hsa_circ_0000231 targets miR-375. For this reason, we further explore the role of *miR-375* in CRC. The mimics-NC and *miR-375* mimics were transfected into SW480 and SW620 cells, respectively, to explore the role of *miR-375* in CRC cells. A CCK-8 assay was performed to detect the effect of *miR-375* on cell proliferation. The results showed that the proliferation ability markedly decreased in the *miR-375* mimics group and in SW480 cells or SW620 cells compared with the control group (*P* < 0.05) (Additional file 8: Fig. S3A, B). The cell cycle detection by flow cytometry revealed that the percentage of G0/G1 cells obviously increased after the overexpression of *miR-375* in SW480 and SW620 cells (Additional file 8: Fig. S3C, D). In addition, the nude mouse model of ectopic tumors has further verified that *miR-375* promotes the proliferation of CRC cells (Additional file 8: Fig. S3E–G).

### *CCND2* was directly targeted by *miR-375* and indirectly regulated by *hsa_circ_0000231*

**Fig. 4:**
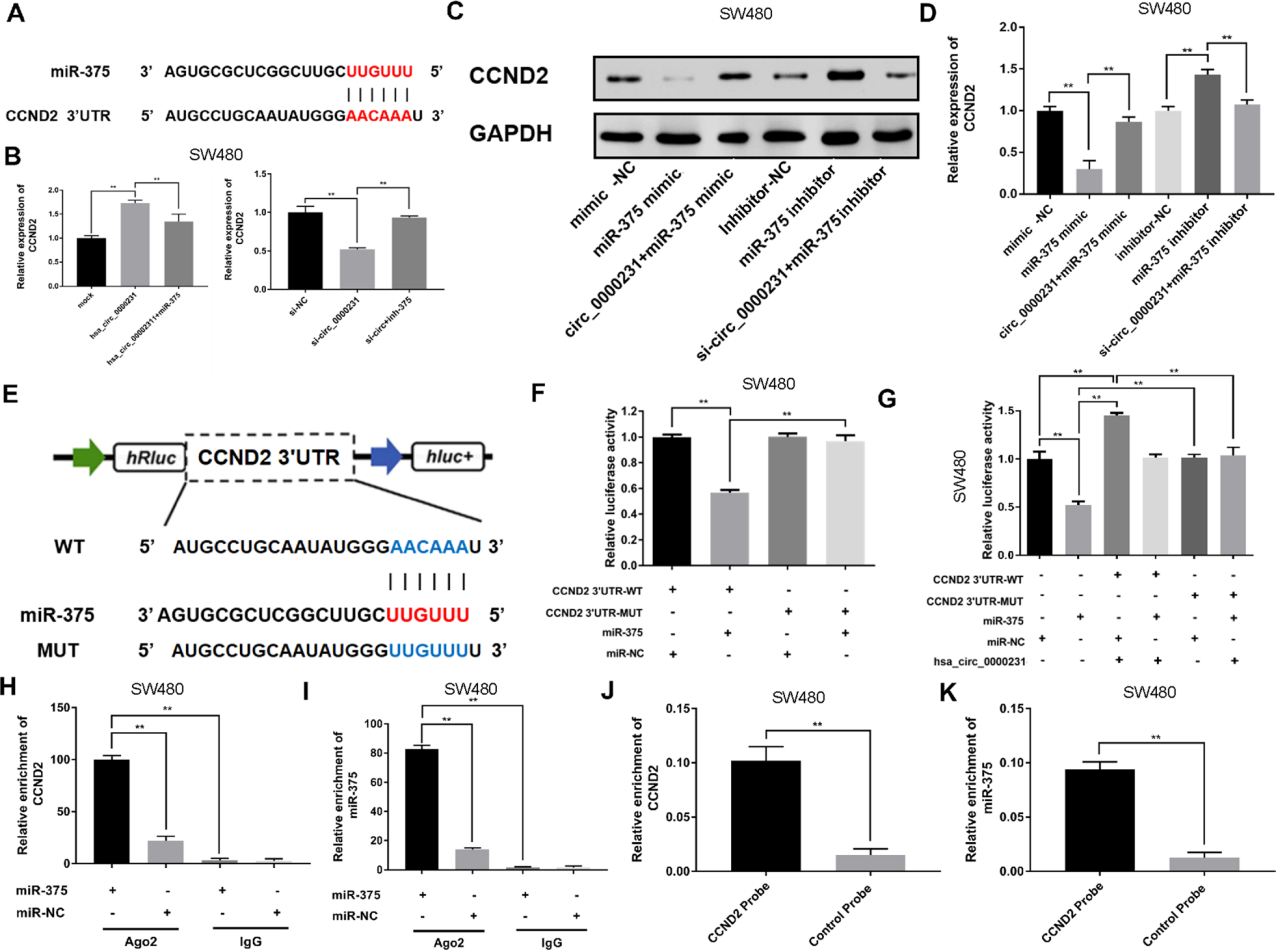
*CCND2* is directly targeted by *miR-375* and indirectly regulated by *hsa_circ_0000231. A* Schematic illustration of *CCND2* 3’UTR-WT and *CCND2* 3’UTR-Mut luciferase reporter vectors. **B** Relative expression of *CCND2* was detected by qRT-PCR in cells transfected with indicated vectors, miRNAs or inhibitors. **C** and **D** Western blot assay was performed to determine the relative expression of *CCND2* transfected with indicated vectors, miRNAs or inhibitors. **E** Schematic illustration of *CCND2* 3’UTR-WT and *CCND2* 3’UTR-Mut luciferase reporter vectors. **F** The relative luciferase activities were detected in SW480 cells after transfected with *CCND2* 3’UTR-WT or *CCND2* 3’UTR-Mut and *miR-375* mimics or miR-NC, respectively. **G** The luciferase activity was recovered after transfection with *hsa_circ_0000231* in the *miR-375* + *CCND2* 3’UTR-WT group. **H** and **I** Anti-AGO2 RIP was executed in SW480 cells after transfection with miR-375 mimic or miR-NC, followed by western blot and qRT-PCR to detect AGO2 protein, *hsa_circ_0000231* and *miR-375*, respectively. **J** and **K** RNA pull-down was executed in SW480 cells, followed by qRT-PCR to detect the enrichment of *hsa_circ_0000231* and *miR-375*. Data were showed as mean ± SD, *p < 0.05, **p < 0.001

According to the TargetScan (http://www.targetscan.org), *CCND2* and *hsa_circ_0000231* shared the same MRE of *miR-375* (Fig. 4A). The present study found that *miR-375* mimics could markedly reduce the expression of *CCND2*, while *miR-375* inhibitors significantly enhanced the level of *CCND2* in SW480 cells. The increase or decrease in *CCND2* expression induced by *miR-375* mimics or inhibitors could be markedly reversed by *hsa_circ_0000231* overexpression or knockdown, respectively, in SW480 cells, as detected by qPCR and Western blot analysis (Fig. 4B–D). These data suggested that *hsa_circ_0000231* could regulate the expression of *CCND2* by serving as a ceRNA for *miR-375* in CRC.

The dual-luciferase reporter assay was conducted to validate the prediction. The results showed that the activity of luciferase reporter vector carrying the *CCND2* 3’-UTR-WT sequence was significantly decreased by *miR-375* mimics compared with the miR-NC groups (Fig. 4E, F). Moreover, the luciferase activity was recovered after transfection with *hsa_circ_0000231* in the *miR-375* + *CCND2* 3’-UTR-WT group (Fig. 4G). Furthermore, an anti-AGO2 RIP assay was performed to detect the relationship between *miR-375* and *CCND2*. The result showed that *miR-375* pull-down by *CCND2* was specifically enriched in SW480 cells (Fig. 4H, I). Then, an RNA pull-down assay was performed in SW480 cells. The analysis demonstrated that endogenous *CCND2* was significantly pulled down by biotinylated probes against *miR-375* (Fig. 4J, K).

### *IGF2BP3* could bind to *hsa_circ_0000231* and *CCND2*, as an RBP to prevent RNA degradation

**Fig. 5:**
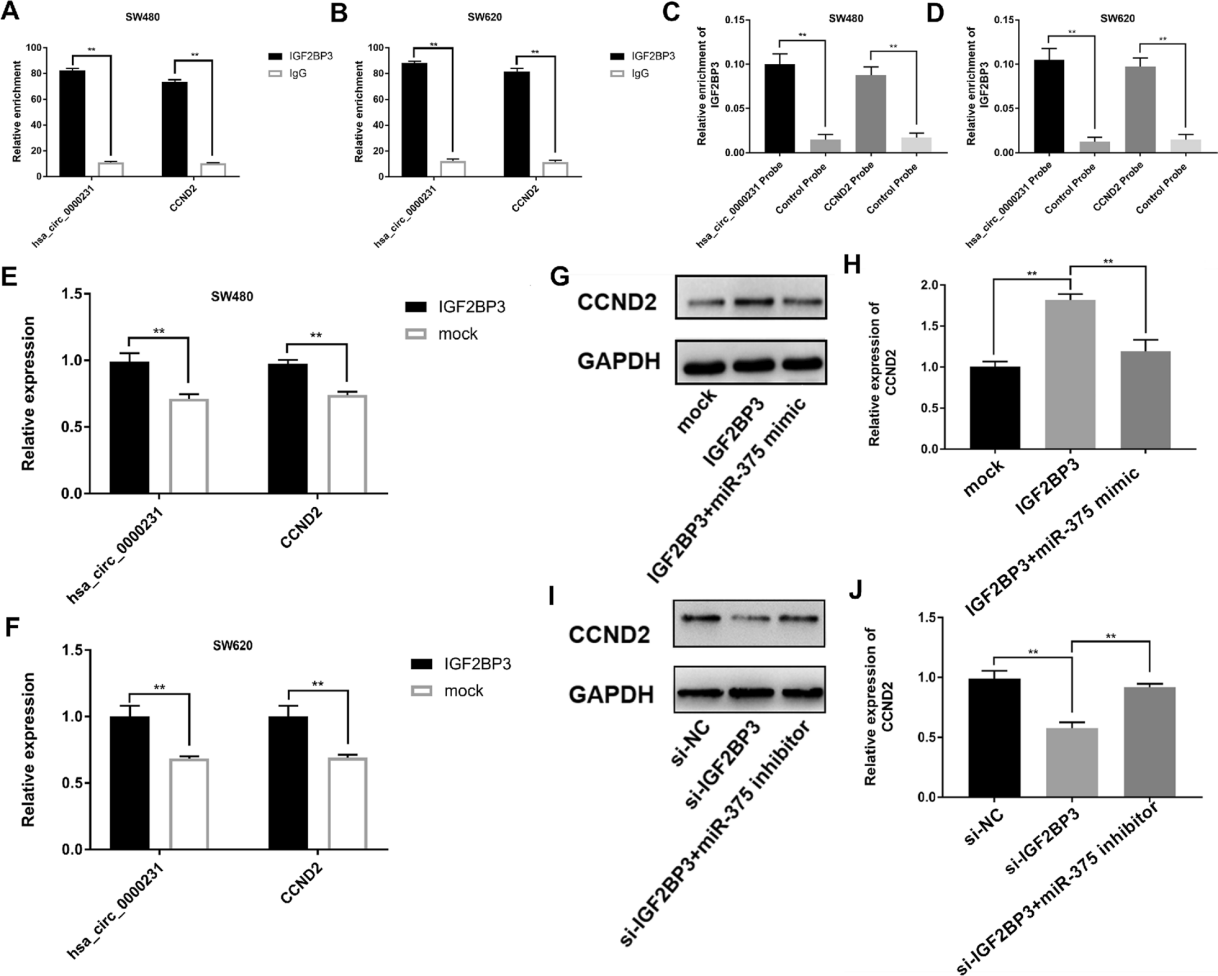
IGF2BP3 could bind to *hsa_circ_0000231* and *CCND2*, as an RBP to protect the stability of RNA. **A** and **B** Anti-IGF2BP3 RIP was executed in SW620 and SW480 cells followed by western blot and qRT-PCR to detect IGF2BP3 protein, *hsa_circ_0000231* and *CCND2*, respectively. **C** and **D** RNA pull-down was executed in SW620 and SW480 cells, followed by qRT-PCR to detect the enrichment of h*sa_circ_0000231* and *CCND2*, respectively. **E** and **F** qRT-PCR was used to detect expression of *hsa_circ_0000231* and *CCND2* after overexpression of IGF2BP3. **G**–**J** Western Blot assay was conducted to confirm the relative expression of CCND2 transfected with indicated vectors, miRNAs or inhibitors in SW480 cells. Data were showed as mean ± SD, *p < 0.05, **p < 0.001

The circBase and Circular RNA interactome databases were used to predict RNA-binding proteins (RBP) that could bind to *hsa_circ_0000231*. *IGF2BP3* had a binding site with *hsa_circ_0000231*, and it could be perfectly matched to the 3'-UTR region of the *CCND2* gene mRNA, which was also the seed sequence region of miR375. An anti-*IGF2BP3* RIP assay was performed to detect the expression of *hsa_circ_0000231* and *CCND2* to verify the combination of *IGF2BP3* with *hsa_circ_0000231* and *CCND2*. The result showed that both *hsa_circ_0000231* and *CCND2* were significantly enriched in SW480 and SW620 cells (Fig. 5A, B). Still, an RNA pull-down assay was conducted in SW480 and SW620 cell lines. The analysis demonstrated that *IGF2BP3* was remarkably pulled down by biotinylated probes against both *hsa_circ_0000231* and *CCND2* (Fig. 5C, D).

*IGF2BP3* was overexpressed to confirm that it exerted a protective effect by increasing the expression of *hsa_circ_0000231* and *CCND2* in CRC cells. The overexpression of *IGF2BP3* could markedly enhance the expressions of *hsa_circ_0000231* and *CCND2* in SW480 and SW620 cells (Fig. 5E, F). These data suggested that *IGF2BP3* might increase the expression of *CCND2* through RBP binding to both *hsa_circ_0000231* and *CCND2* in CRC. Moreover, Western blot analysis was conducted to confirm that the upregulation or downregulation of *IGF2BP3* markedly enhanced or decreased the expression of *CCND2*, and the effect of *IGF2BP3* could be partially reversed by mimics or inhibitor of *miR-375* in SW480 and SW620 cells (Fig. 5G–J).

### *Hsa_circ_0000231* promoted CRC proliferation through the *hsa_circ_0000231*/*IGF2BP3*/*miR-375*/*CCND2* axis

**Fig. 6:**
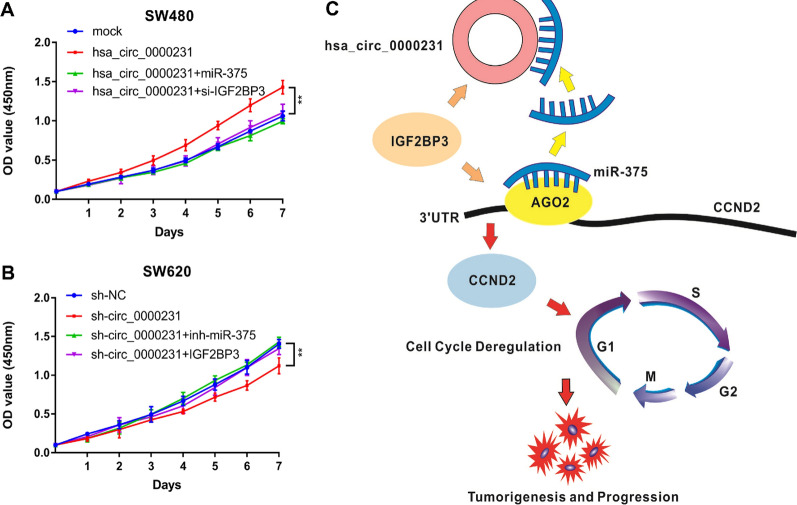
*hsa_circ_0000231* promotes CRC proliferation through *hsa_circ_0000231/IGF2BP3/miR-375/CCND2* axis. **A** and **B** The cell proliferations were determined after transfection with indicated vectors, miRNAs or inhibitors by CCK-8 assays, respectively. **C** Schematic diagram of how *hsa_circ_0000231* promotes CRC tumorigenesis and progression. Data were showed as mean ± SD, *p < 0.05, **p < 0.001

Rescue experiments were applied using *miR-375* mimics and inhibitors to ensure whether *hsa_circ_0000231* executed its biological function through the *hsa_circ_0000231*/*IGF2BP3*/*miR-375*/*CCND2* axis. The results indicated that the *miR-375* mimics reversed the proliferation-promoting effects of *hsa_circ_0000231* overexpression in SW480 cells, whereas *miR-375* inhibitors could rescue the proliferation-suppressing effects of the knockdown of *hsa_circ_0000231* in SW620 cells, as detected by the CCK8 assay. The effects caused by an increase or decrease in the expression of *hsa_circ_0000231* could be partially rescued by si-*IGF2BP3* or overexpression of *IGF2BP3* in SW480 and SW620 cells, respectively (Fig. 6A, B). In summary, these data demonstrated that *hsa_circ_0000231* might serve as a ceRNA for *miR-375* and was protected by *IGF2BP3* to regulate *CCND2* expression (Fig. 6C).

## Discussion

The circRNA is abnormally expressed in various cancers, such as esophageal cancer [15], gastric cancer [16], pancreatic cancer [17], breast cancer [18] and liver cancer [19]. It is associated with cell proliferation, invasion, and metastasis, and even patient prognosis. The reports on the role of circRNA in CRC are mostly related to the relationship between circRNA and clinicopathological indicators. A few in-depth studies were performed on molecular regulation mechanisms. Related research found that circ0000069 promotes the proliferation, invasion, and migration of CRC cells and was associated with age, lymph node metastasis, and TNM staging in patients with CRC [20]. He et al. found that *circRNA_103948* regulates the autophagy of colorectal cancer cells through the *miR-1236-3p/TPT1* axis and affects the progression of CRC [21]. In addition, studies have found that *Circ-GALNT16* inhibits the progression of CRC by regulating the sequence-specific DNA binding capacity of the *hnRNPK-p53* transcription complex mediated by *SENP2* to inhibit Serpine1 expression [22]. These studies show that circRNA plays an important role in the occurrence and development of colorectal cancer. Therefore, it is urgent to dig into valuable circRNA and explore its regulatory mechanism.

With the development of sequencing technology, it is increasingly used in scientific research, including the medical field. Therefore, this study used high-throughput sequencing technology to analyze the differentially expressed circRNAs in CRC and adjacent normal tissues. The results show that *hsa_circ_0000231* is highly expressed in CRC (Fig. 1B). Studies have found that *hsa_circ_0000231* affects the progression of colorectal cancer by targeting *miR-622* and *miR-502-5p* [23, 24]. The results of this study also found that overexpression of *hsa_circ_0000231* can promote the growth of CRC cells and lead to a poor prognosis (Figs. 1G, H, 2I, K), which is consistent with previous studies. However, whether *hsa_circ_0000231* has other mechanisms to regulate the progression of colorectal cancer is still unknown.

Increasing evidence indicated that some circRNAs could serve as sponges for miRNAs to regulate the expression of miRNA target genes in multiple human diseases [25], including colorectal cancer. For example, hsa_circ_001680 promoted CRC development by functioning as a sponge for miR-340 to influence the expression of BMI1 [26]. Thus, fully understanding how *hsa_circ_0000231* functioned in CRC would provide a novel insight into the oncogenesis mechanism. The present study found using bioinformatics analysis that *hsa_circ_0000231* contained the MRE of *miR-375*. *Hsa_circ_0000231* and *miR-375* were co-located in the cytoplasm of CRC cells and tissues. Further, the dual-luciferase reporter, anti-AGO2 RNA immunoprecipitation, and RNA pull-down assays confirmed that *hsa_circ_0000231* could interact with *miR-375* directly, indicating that circ_0000231 exerted an oncogenic effect via sponging *miR-375* in CRC. Consistent with the results of the present study, *miR-375* was significantly downregulated in CRC tissues and cell lines and negatively correlated with the degree of malignancy of CRC [27]. The findings of this study indicated that *hsa_circ_0000231* served as an oncogene by sponging *miR-375* in CRC, revealing the significance of interaction between *hsa_circ_0000231*, and *miR-375* in the tumorigenesis and development of CRC.

According to the ceRNA hypothesis, circRNA could act as a ceRNA to modulate the expression of miRNA target genes. *CCND2*, a vital cell cycle regulator, and *hsa_circ_0000231* were co-overexpressed in CRC, further confirming that the cell cycle was closely related to the tumorigenesis and development of CRC [28]. Moreover, the bioinformatics analysis indicated using miRcode and TargetScan that *CCND2* was one of the potential targets of *miR-375*. Next, a dual-luciferase reporter assay confirmed that *miR-375* could directly target the 3′-UTR of *CCND2*. Additionally, the upregulation of *miR-375* led to the knockdown of *CCND2* at the mRNA and protein levels, whereas the downregulation of *miR-375* had an opposite effect. *CCND2* mainly regulates the cell cycle progression [29]. The downregulation of *hsa_circ_0000231* resulted in G1/S-phase cell cycle arrest. *CCND2* was involved in determining progression through checkpoints in G1/S and G2/M phases that dictated whether a cell could proceed with DNA replication and cell division. The data suggested that *hsa_circ_0000231* knockdown might inhibit the expression of *CCND2*, leading to decreased activity and G1/S-phase cell cycle arrest. The dysregulation of *CCND2* activity was implicated in multiple cancers, including CRC [28].

Consistent with previous findings, the present study found that *CCND2* was significantly regulated in CRC tissues and the overexpression of *CCND2* correlated with shorter OS. This study showed that the overexpression of *hsa_circ_0000231* could increase the expression of *CCND2* at both mRNA and protein levels, while the knockdown of *hsa_circ_0000231* exhibited a reverse effect, thus validating the crosstalk between *hsa_circ_0000231* and *CCND2*. Furthermore, these effects could be partially abolished by *miR-375* mimics or inhibitors, hence supporting the hypothesis that *hsa_circ_0000231* functioned as a ceRNA to promote *CCND2*-mediated proliferation via decoying *miR-375* in CRC.

Besides, RBP is vital in the regulation of post-transcriptional gene expression, and circRNA may act as an RBP "super sponge" by binding to RBP, thus changing the splicing pattern or mRNA stability [30–32]. In the present study, a circular RNA interactome database was used to forecast that *IGF2BP3* might combine with *hsa_circ_0000231* via RBA-protein-binding sites. Another study found through starBase database [33] prediction that *IGF2BP3* also combined with *CCND2*. *IGF2BP3* is a member of the RBP family and functions in many biological processes, such as mRNA localization, translation, and stability maintenance [34]. Previous studies found that the expression of *IGF2BP3* was associated with the occurrence, metastasis, and poor prognosis of malignant tumors [35]. Regarding the regulatory effect of *IGF2BP3* on target genes, some studies suggested that the *IGF2BP3* protein bound to the 3’-UTR of target gene mRNA and compete with miRNA for binding sites, thus protecting the mRNA of target genes from miRNA degradation, including maintaining the stability of *CCND2* mRNA and promoting *CCND2* translation [36, 37].

Therefore, it was speculated that *IGF2BP3* could enhance the stability of *hsa_circ_0000231* and protect *CCND2* from degradation. To confirm a previous suggestion, it was presumed that *IGF2BP3* might bind to *hsa_circ_0000231* and *CCND2* at the same time, and the binding site was consistent with the binding site of *miR-375* and *CCND2*. RIP, RNA pull-down, qRT-PCR, and Western blot assays were further performed to confirm that the expression of hsa_circ_0091073 and *CCND2* could be upregulated by increasing the expression of *IGF2BP3*, while the expression of hsa_circ_0091073 and *CCND2* was downregulated by interfering with the expression of the *IGF2BP3* gene. Meanwhile, *IGF2BP3* protected *CCND2* from the regulation of *miR-375*, and the regulatory effect of *miR-375* on *CCND2* was opposite to that of *IGF2BP3*.vTherefore, *IGF2BP3* might not only bind to *hsa_circ_0000231* but also inhibit the degradation of *CCND2* mRNA by *miR-375* at the 3’-UTR binding site of *CCDN2* mRNA, thus maintaining the stability of *CCND2* mRNA.

However, this study also has some limitations. First, this study found that *hsa_circ_0000231* was significantly highly expressed in CRC. When verifying the function of *hsa_circ_0000231*, this study only explored the effect of knocking down *hsa_circ_0000231* on the phenotype of CRC cells. However, when conducting in vivo experiments in nude mice, we used cell lines overexpressing *hsa_circ_0000231* for experiments, which partially supplemented this limitation. Secondly, this study found that the expression of other cell cycle genes was also inhibited after knocking down *hsa_circ_0000231*, but no further research was carried out, and further analysis may be needed in the future. Finally, this study only explores the ceRNA network mechanisms when exploring the regulatory mechanism of *hsa_circ_0000231*, and it may be necessary to further explore it from other aspects in order to discover new regulatory mechanisms.

## Conclusions

This study was novel in demonstrating that *hsa_circ_0000231* might sponge *miR-375* to modulate *CCND2* expression, and *IGF2BP3* could protect *hsa_circ_0000231* and *CCND2*, leading to the tumorigenesis and development of CRC. The regulatory network involving the *hsa_circ_0000231*/*IGF2BP3*/*miR-375*/*CCND2* axis might provide a better understanding of the potential mechanism underlying the pathogenesis and progression of CRC. *hsa_circ_0000231* could be a valuable prognosis marker and a promising diagnostic and therapeutic target for CRC in the future.

## Supplementary Information

**Additional file 1: Table S1.** List of primers sequences used for real-time PCR.

**Additional file 2: Table S2.** List of antibodies used for Western Blot.

**Additional file 3: Table S3.** Relationship between *has_circ_0000231* and *CCND2* expression and clinicopathological data.

**Additional file 4: Table S4.** Univariate and multivariate Cox regression analysis of *has_circ0000231* and survival in patients with CRC.

**Additional file 5: Table S5.** Classification of circRNA.

**Additional file 6: Figure S1.** Transfection of *si-circ_0000231* inhibits the expression of *hsa_circ_0000231* in SW480 cells. The results showed that the expression of *hsa_circ_0000231* in the siRNA group was significantly lower than that in the si-NC group (*p* < 0.05).

**Additional file 7: Figure S2.** *CCND2* might be a downstream target of hsa_circ_0000231 in CRC. A 10 mostly decreased genes were shown after downregulation of hsa_circ_0000231. B qRT-PCR assay was used to verify the results of PCR assay (The fold change is the absolute value). C qRT-PCR was used to detect expression of *CCND2* after interference with hsa_circ_0000231. D Relative expression of *CCND2* in CRC tissues (CRT) and adjacent normal tissues (CRN) was determined by qRT-PCR (n = 160). E Kaplan–Meier survival curve of overall survival in 160 patients with CRC according to the *CCND2* expression. Patients were divided into high expression and low expression group by median expression. F Pearson correlation analysis of *hsa_circ_0000231* and *CCND2* expression in 160 CRC tissues. G and H. Western blot assay was performed to reveal the expression of *CCND2* in xenograft tumors.

**Additional file 8: Figure S3.** *miR-375* suppresses tumor growth of CRC cells in vitro and in vivo*.* A and B. The growth curves of cells transfected with indicated vectors were evaluated by CCK8 assays. C and D. The cell cycle progression was conducted by flow cytometry after overexpression of miR-375. Data were showed as mean ± SD, *p < 0.05, **p < 0.001. E. Images of xenograft tumors of each group (n = 5). F. The difference in tumor volume in different intervention groups. *p < 0.05, **p < 0.001. G. The difference in tumor weight in different intervention groups. *p < 0.05, **p < 0.001.

## Data Availability

All materials and data are available, and we are very willing to provide corresponding help to researchers for non-commercial research.

## Abbreviations

circRNA: Circular RNA
CRC: Colorectal cancer
ceRNA: Competing endogenous RNA
*CCND2*: Cyclin D2
MRE: MiRNA response element
miRNA: MicroRNA
siRNA: Small interfering RNA
RIP: RNA immunoprecipitation
AGO2: Anti-Argonaute2
RBP: RNA binding protein
