# Supplementary material for: hsa_circ_0000231 Promotes colorectal cancer cell growth through upregulation of CCND2 by IGF2BP3/miR-375 dual pathway
Source: Cancer Cell Int. 2022 Jan 15;22:27. doi: 10.1186/s12935-022-02455-8 (PMC8760675; doi:10.1186/s12935-022-02455-8)
Supplement: Supplementary file 1 — Additional file 1: Table S1. List of primers sequences used for real-time PCR. [file 12935_2022_2455_MOESM1_ESM.docx]

**Table S1 List of primer sequences used for real-time PCR**

| Gene | Primer Sequences |
| --- | --- |
| has_circ_0000231 | F:5’GATTCCGCAGGAGAAGGCTC3’ |
|  | R:5’GGCTTTATGGCTTGTTGGATGA3’ |
| CCND2 | F:5’TCCTGGCCTCCAAACTCAAA3’ |
|  | F:5’AAGTCATGAGGAGTGACAGC3’ |
| IGF2BP3 | F:5’TATATCGGAAACCTCAGCGAGA3’ |
|  | R:5’GGACCGAGTGCTCAACTTCT3’ |
| GAPDH | F:5’-CACCCACTCCTCCACCTTTG3’ |
|  | R:5’-CCACCACCCTGTTGCTGTAG3’ |
