# Supplementary material for: hsa_circ_0000231 Promotes colorectal cancer cell growth through upregulation of CCND2 by IGF2BP3/miR-375 dual pathway
Source: Cancer Cell Int. 2022 Jan 15;22:27. doi: 10.1186/s12935-022-02455-8 (PMC8760675; doi:10.1186/s12935-022-02455-8)
Supplement: Supplementary file 2 — Additional file 2: Table S2. List of antibodies used for Western Blot. [file 12935_2022_2455_MOESM2_ESM.docx]

**Table S2 List of antibodies used for Western Blot**

| **Antibody** | | | | |  |  |
| --- | --- | --- | --- | --- | --- | --- |
| **Name** | Origin | Conjugation | Corporation | Cat# | Application |  |
| **CCND2** | Polyclonal Rabbit | Unconjugated | Abcam | Ab230883 | WB, ICC/IF |  |
| **GAPDH** | Monoclonal Mouse | Unconjugated | Cell Signaling Technology | 9139 | WB |  |
