# Supplementary material for: hsa_circ_0000231 Promotes colorectal cancer cell growth through upregulation of CCND2 by IGF2BP3/miR-375 dual pathway
Source: Cancer Cell Int. 2022 Jan 15;22:27. doi: 10.1186/s12935-022-02455-8 (PMC8760675; doi:10.1186/s12935-022-02455-8)
Supplement: Supplementary file 3 — Additional file 3: Table S3. Relationship between has_circ_0000231 and CCND2 expression and clinicopathological data. [file 12935_2022_2455_MOESM3_ESM.docx]

**Table S3 Relationship between has_circ_0000231 and CCND2 expression and clinicopathological data**

| clinicopathological features | | expression of hsa_circ_0000231 | | P value | expression of CCND2 | | P value |
| --- | --- | --- | --- | --- | --- | --- | --- |
|  |  | Low | High |  | Low | High |  |
| Gender |  |  |  | 0.791 |  |  | 0.262 |
|  | Male | 44 | 39 |  | 48 | 43 |  |
|  | Female | 36 | 41 |  | 32 | 37 |  |
| Age at diagnosis |  |  |  | 0.162 |  |  | 0.715 |
|  | ≤ 60 | 19 | 27 |  | 21 | 19 |  |
|  | ＞60 | 61 | 53 |  | 59 | 61 |  |
| Tumor size (cm) |  |  |  | **0.032*** |  |  | 0.260 |
|  | ≤ 5 | 18 | 8 |  | 14 | 9 |  |
|  | ＞5 | 62 | 72 |  | 66 | 71 |  |
| Location |  |  |  | 0.733 |  |  | 0.391 |
|  | proximal | 26 | 24 |  | 22 | 27 |  |
|  | distal | 54 | 56 |  | 58 | 53 |  |
| Differentiation |  |  |  | 0.750 |  |  | 0.078 |
|  | Well-moderate | 44 | 46 |  | 41 | 52 |  |
|  | Poor | 36 | 34 |  | 39 | 28 |  |
| Depth of invasion |  |  |  | 0.122 |  |  | **0.032*** |
|  | T1-T2 | 13 | 21 |  | 8 | 18 |  |
|  | T3-T4 | 67 | 59 |  | 72 | 62 |  |
| Lymph node metastasis |  |  |  | **0.002*** |  |  | 0.514 |
|  | No | 7 | 22 |  | 11 | 14 |  |
|  | Yes | 73 | 58 |  | 69 | 66 |  |
| TNM stage |  |  |  | **0.015*** |  |  | **0.043*** |
|  | I-II | 9 | 21 |  | 16 | 7 |  |
|  | III-IV | 71 | 59 |  | 64 | 73 |  |
| distant metastasis |  |  |  | 0.276 |  |  | 0.514 |
|  | No | 65 | 70 |  | 69 | 66 |  |
|  | Yes | 15 | 10 |  | 11 | 14 |  |
|  |  |  |  |  |  |  |  |
