# Supplementary material for: hsa_circ_0000231 Promotes colorectal cancer cell growth through upregulation of CCND2 by IGF2BP3/miR-375 dual pathway
Source: Cancer Cell Int. 2022 Jan 15;22:27. doi: 10.1186/s12935-022-02455-8 (PMC8760675; doi:10.1186/s12935-022-02455-8)
Supplement: Supplementary file 4 — Additional file 4: Table S4. Univariate and multivariate Cox regression analysis of has_circ0000231 and survival in patients with CRC. [file 12935_2022_2455_MOESM4_ESM.docx]

**Table S4 Univariate and multivariate Cox regression analysis of has_circ0000231 and survival in patients with CRC**

| Clinical variables | Univariate analysis | | *p* | Multivariate analysis | | *p* |
| --- | --- | --- | --- | --- | --- | --- |
|  | HR | 95%Cl |  | HR | 95%CL |  |
| Age (≥50 vs. < 50) | 0.952 | 0.415–1.921 | 0.814 |  |  |  |
| T stage (T1/T2 vs T3/T4) | 1.722 | 1.012–3.021 | **0.026*** |  |  |  |
| N stage (N0 vs. Nx) | 2.853 | 0.442–5.714 | **0.011*** |  |  |  |
| TNM stage (I-II vs. III-IV) | 2.296 | 1.141–4.617 | **0.020*** | 3.092 | 1.221–6.102 | **0.015*** |
| has_irc_0000231 (low vs. high) | 4.652 | 2.866-15.108 | **0.012*** | 5.042 | 2.604–16.105 | **< 0.001*** |

Abbreviations: HR hazard ratio, CI confidence interval **p* < 0.05
