# Supplementary material for: hsa_circ_0000231 Promotes colorectal cancer cell growth through upregulation of CCND2 by IGF2BP3/miR-375 dual pathway
Source: Cancer Cell Int. 2022 Jan 15;22:27. doi: 10.1186/s12935-022-02455-8 (PMC8760675; doi:10.1186/s12935-022-02455-8)
Supplement: Supplementary file 5 — Additional file 5: Table S5. Classification of circRNA. [file 12935_2022_2455_MOESM5_ESM.docx]

**Table S5. Classification of circRNA**

| **Style** | **Up-regulation (quantity/proportion)** | **Down-regulation**  **(quantity/proportion)** |
| --- | --- | --- |
| **CircRNA (exonic)** | 256 / (92.09%) | 129 / (87.76%) |
| **CircRNA (intronic)** | 8 / (2.88%) | 12 / (8.16%) |
| **CircRNA (antisense)** | 3 / (1.07%) | 3 / (2.04%) |
| **CircRNA (intragenic)** | 11 / (3.96%) | 3 / (2.04%) |
| **Summary** | **278 / (100%)** | **147 / (100%)** |
