# Supplementary figures and images for: hsa_circ_0000231 Promotes colorectal cancer cell growth through upregulation of CCND2 by IGF2BP3/miR-375 dual pathway
Source: Cancer Cell Int. 2022 Jan 15;22:27. doi: 10.1186/s12935-022-02455-8 (PMC8760675; doi:10.1186/s12935-022-02455-8)

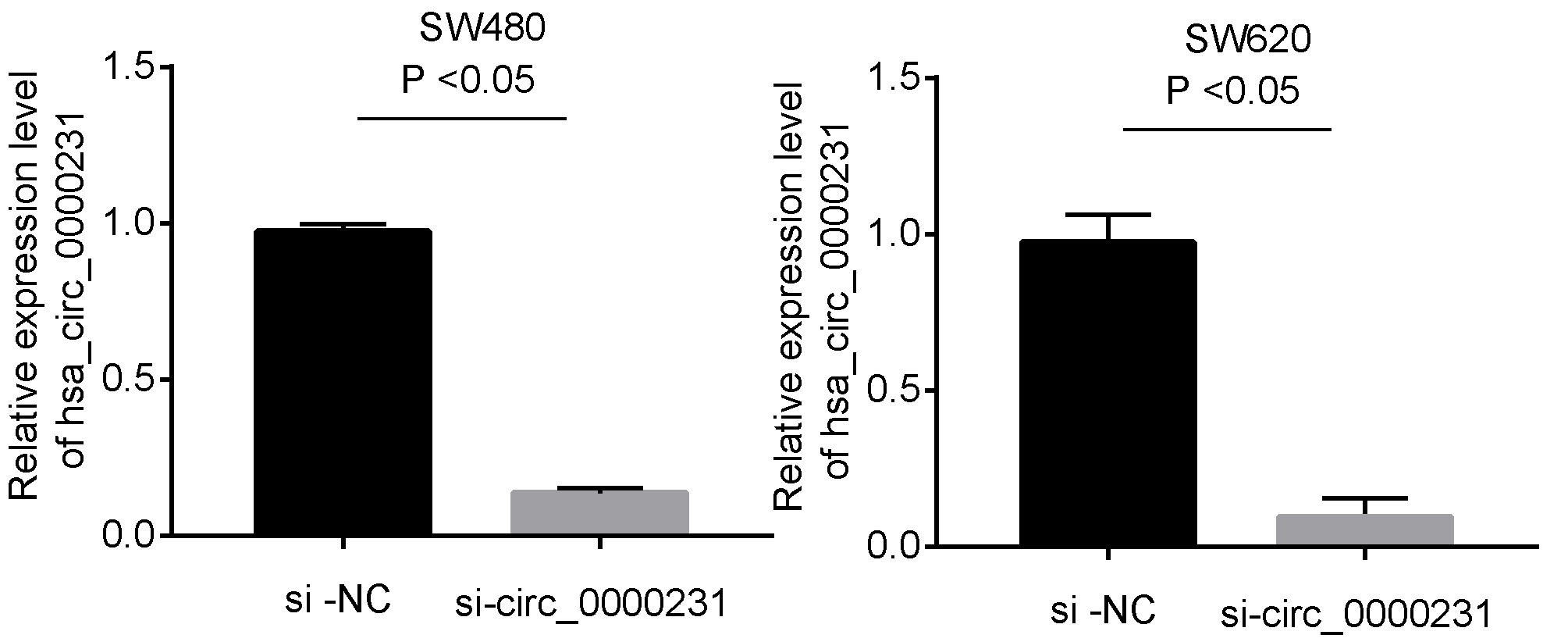

Supplement: Supplementary file 6 — Additional file 6: Figure S1. Transfection of si-circ_0000231 inhibits the expression of hsa_circ_0000231 in SW480 cells. The results showed that the expression of hsa_circ_0000231 in the siRNA group was significantly lower than that in the si-NC group (p < 0.05). [file 12935_2022_2455_MOESM6_ESM.tif]

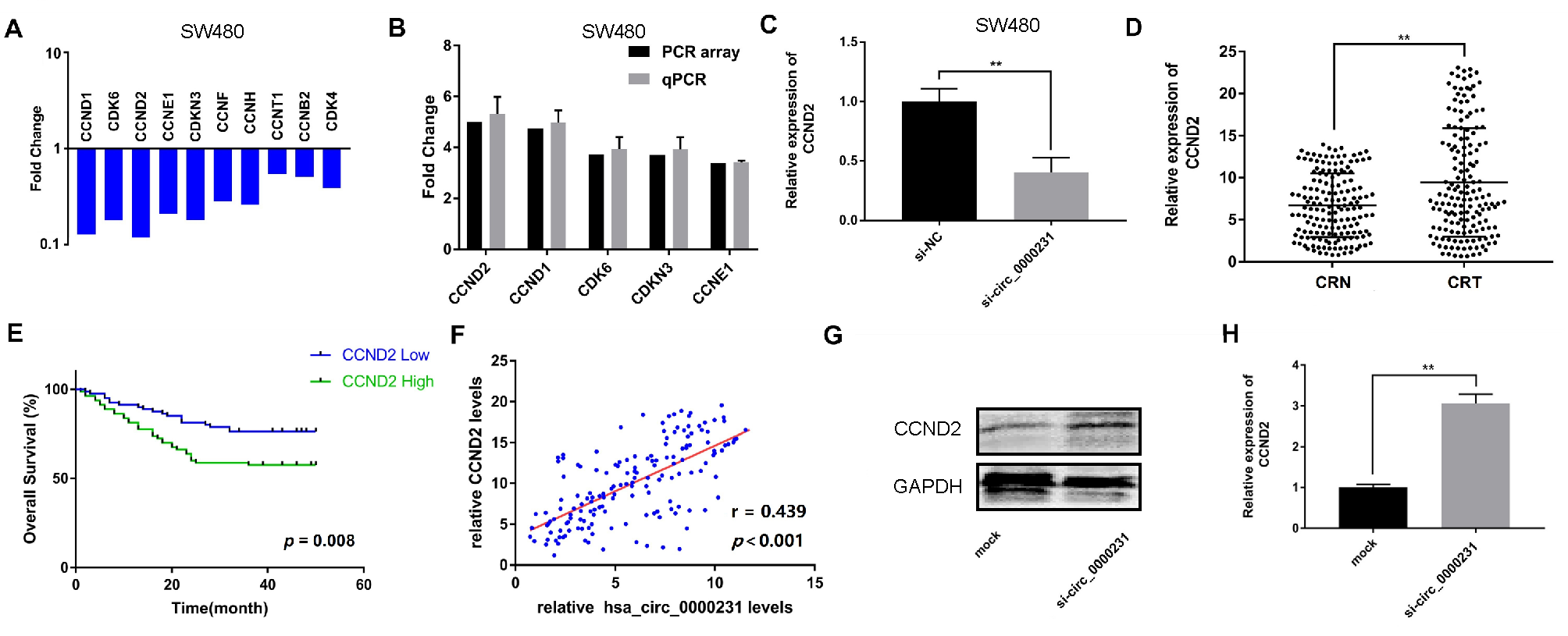

Supplement: Supplementary file 7 — Additional file 7: Figure S2. CCND2 might be a downstream target of hsa_circ_0000231 in CRC. A 10 mostly decreased genes were shown after downregulation of hsa_circ_0000231. B qRT-PCR assay was used to verify the results of PCR assay (The fold change is the absolute value). C qRT-PCR was used to detect expression of CCND2 after interference with hsa_circ_0000231. D Relative expression of CCND2 in CRC tissues (CRT) and adjacent normal tissues (CRN) was determined by qRT-PCR (n = 160). E Kaplan–Meier survival curve of overall survival in 160 patients with CRC according to the CCND2 expression. Patients were divided into high expression and low expression group by median expression. F Pearson correlation analysis of hsa_circ_0000231 and CCND2 expression in 160 CRC tissues. G and H. Western blot assay was performed to reveal the expression of CCND2 in xenograft tumors. [file 12935_2022_2455_MOESM7_ESM.tif]

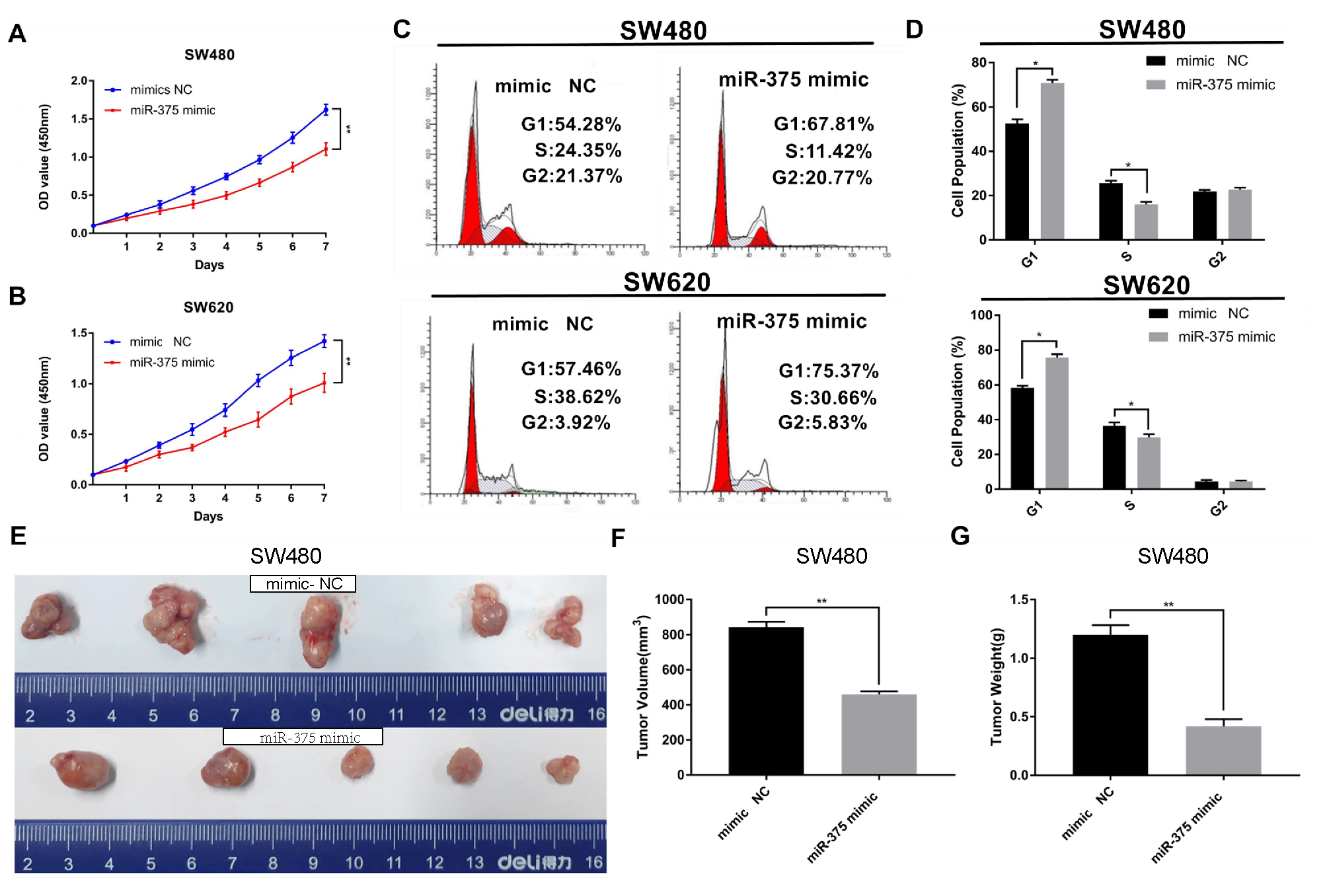

Supplement: Supplementary file 8 — Additional file 8: Figure S3. miR-375 suppresses tumor growth of CRC cells in vitro and in vivo. A and B. The growth curves of cells transfected with indicated vectors were evaluated by CCK8 assays. C and D. The cell cycle progression was conducted by flow cytometry after overexpression of miR-375. Data were showed as mean ± SD, *p < 0.05, **p < 0.001. E. Images of xenograft tumors of each group (n = 5). F. The difference in tumor volume in different intervention groups. *p < 0.05, **p < 0.001. G. The difference in tumor weight in different intervention groups. *p < 0.05, **p < 0.001. [file 12935_2022_2455_MOESM8_ESM.tif]
